# Influence of Adjacent Teeth Absence or Extraction on the Outcome of Non-Surgical Periodontal Therapy

**DOI:** 10.3390/ijerph16224344

**Published:** 2019-11-07

**Authors:** Jia-Hong Lin, Che-Chang Tu, Yi-Wen Chen, Chen-Ying Wang, Cheing-Meei Liu, Mark Yen-Ping Kuo, Po-Chun Chang

**Affiliations:** 1 Division of Periodontics, Department of Dentistry, National Taiwan University Hospital, Taipei 100, Taiwan; b00402010@ntu.edu.tw (J.-H.L.); d05422002@ntu.edu.tw (C.-C.T.); evelyn.peri@gmail.com (Y.-W.C.); jybean@gmail.com (C.-Y.W.); cmsliu@ntu.edu.tw (C.-M.L.); oddie@ntu.edu.tw (M.Y.-P.K.); 2 Department of Dentistry, School of Dentistry, National Taiwan University, Taipei 100, Taiwan; 3 Graduate Institute of Clinical Dentistry, School of Dentistry, National Taiwan University, Taipei 100, Taiwan

**Keywords:** tooth extraction, periodontitis, periodontal pocket, root planing, molar

## Abstract

*Background*: Extraction of periodontally compromised or strategically non-important teeth is often an integral part of non-surgical periodontal treatment (NSPT). This study evaluated the association between the status of adjacent teeth and the outcome of NSPT on molars. *Methods*: Charting data of patients with generalized chronic periodontitis receiving NSPT in 2012–2014 were included. The association between initial clinical parameters and significant clinical improvement, including the reductions of probing pocket depth (PPD) and clinical attachment loss (CAL), in molar teeth with severe periodontitis after NSPT was assessed by a generalized linear model and logistic regression. *Results*: ≥7 mm PPD and <2 mm gingival recession (REC) at the tooth level, and ≥7 mm PPD, ≥7 mm CAL and <2 mm REC at the site level, were associated with significant clinical improvement. Absence or extraction of an adjacent tooth achieved an additional 0.22–0.23 mm and 0.60–0.83 mm clinical improvement. Among the interproximal sites, ≥7 mm PPD, <2 mm REC, ≥7 mm CAL, <Degree II furcation involvement, and absence of an adjacent tooth were associated with significant clinical improvement. *Conclusion*: Absence or extraction of teeth during NSPT significantly improves the PPD and CAL of the adjacent periodontal sites of molars.

## 1. Introduction

Periodontitis is a chronic infectious disease of the periodontium that affects over 50% of the adult population in Taiwan [1]. Non-surgical periodontal therapy (NSPT), including oral hygiene instruction, scaling, and root planing, has been proposed as initial treatment for periodontitis. The objective of NSPT is to control the microbial etiological factors to halt disease progression and render an acceptable and healthy periodontal condition [2]. As Cobb reported, a 1–3 mm reduction of probing pocket depth (PPD) and a 0–2 mm reduction of CAL can be expected following NSPT [3]. Several guidelines have been established, based on large-scale evaluation of the interaction between clinical periodontal parameters and disease progression, to predict the prognosis of periodontitis-affected teeth and subsequent treatment planning [2,4,5,6,7].

When a prognosis of “hopeless tooth” is determined, the next question is the timing of extraction. It has been suggested that the hopeless tooth should be extracted unless the acute symptoms and signs presented could be temporarily retained to maintain posterior occlusal stops and anterior esthetics [2]. A previous study reported that retention of a hopeless tooth led to 10 times faster alveolar bone loss rate at neighboring teeth over 2 years compared with the extraction of a hopeless tooth [8]. Following a limited hygiene phase, Grassi et al. [9] reported that tooth extraction resulted in a 0.5–1.5 mm PPD reduction. Compared to those without tooth extraction, an additional 0.60 mm PDD reduction and 0.26 mm CAL reduction was noted on the adjacent sites with initial 4–9 mm PPD, whereas the radiographic bone level and oral hygiene performance were altered to a limited extent.

Molars are characterized by complex root anatomy that causes difficulty in adequate cleaning. Compared with non-molar teeth, PPD and CAL reductions were inferior on molars after NSPT, specifically for sites with 4–6 mm initial PPD [10]. Wylam et al. [11] reported that residual plaque and calculus were detected on 54.3% of root surfaces of molars after NSPT, and the presence of deposits was even higher (93.2%) in the furcational area. Parashis et al. also indicated that the effectiveness of NSPT on molars was significantly reduced when ≥7 mm PPD or furcation involvement (FI) was present [12].

To improve the efficiency of NSPT on molars, in this present study, we hence sought to investigate whether the presence or absence of adjacent teeth affects the outcome of NSPT specifically on severely periodontitis-affected (CAL ≥ 5 mm) molars. A large-scale retrospective study (>200 patients) was conducted to answer two focus questions: whether sites without adjacent teeth would achieve greater improvement after NSPT and whether tooth extraction would grant better outcomes of NSPT at adjacent sites.

## 2. Materials and Methods

### 2.1. Ethical Approval and Study Population

This retrospective study was conducted at the Department of Dentistry, National Taiwan University Hospital (NTUH) under an approved protocol (No. 201501034RINB) from the Institutional Review Board of NTUH, in accordance with the Helsinki Declaration of 1975, as revised in 2008. A total of 275 adult patients (Chinese Han ethnic population) aged between 20 and 75 years, who had completed full-mouth NSPT under the supervision of CPC, CYW, and YWC at the Department of Dentistry, NTUH from January 2012 to 2014, were included. NSPT included full-mouth scaling and root planing (ScRP) completed in two to three appointments, oral hygiene instruction and reinforcement in two to four appointments, and re-evaluation of full-mouth periodontal conditions after 4–8 weeks of ScRP. The exclusion criteria were: (1) had fewer than 16 teeth at re-evaluation of NSPT; (2) the initial periodontal status did not fulfill the definition of generalized chronic periodontitis according to the classification defined by the American Academy of Periodontology in 1999 and updated in 2015 [13]; (3) was recorded as a smoker during NSPT; (4) no record of systemic diseases affecting periodontal health or wound healing; (5) had a record of taking antibiotics, anti-inflammatory drugs, or drugs affecting wound healing during the course of NSPT.

### 2.2. Clinical Data Collection

Clinical parameters, including PPD, CAL, gingival recession (REC), keratinized mucosa (KM), dichotomous plaque score (PS) [14], tooth mobility (MB) [2], and gingival bleeding index (GBI) [15] before NSPT and at the time of re-evaluation, were retrieved from the dental charting form of NTUH. PPD, REC, and CAL were measured at six surfaces of each tooth, and PS and GBI were measured at four surfaces of each tooth. FI was recorded immediately before NSPT and measured at two to three regions of molars based on the root anatomy [16]. All clinical parameters were examined using a manual periodontal probe (PCP10-SE, Hu-Friedy Co. Inc., Chicago, IL, USA), except for FI, which was assessed by a Nabers probe (Q-2N, Hu-Friedy Co. Inc., Chicago, IL, USA), and PS, which was assessed by erythrosine-containing disclosing agent (Beauteeth Co. Ltd., New Taipei City, Taiwan).

### 2.3. Statistical Analysis

Data from sites with ≥5 mm CAL of permanent molars were analyzed individually (site level). Tooth-level analysis was based on the mean of all examined sites of the same tooth with at least one eligible site. The examined teeth and sites were equally dichotomized to “high responder” (HR) and “low responder” (LR) based on the cut-off value, which referred to the medians of the change of parameters (PPD and CAL) from all examined teeth or sites respectively. All clinical parameters were expressed as means ± standard deviations (SD) with the interquartile range (IQR). Because the examined clinical parameters were not assumed to exhibit a normal distribution, the Mann-Whitney test was used to compare the difference between groups, with a *p* value less than 0.05 considered statistically significant.

To evaluate the influence of adjacent tooth absence or extraction, the examined sites were further stratified based on the presence or absence of adjacent teeth at initial assessment, or the extraction or preservation of adjacent teeth during NSPT. Generalized linear modeling (GLM) and logistic regression were used to compare the differences in distribution between outcome parameters (PPD and CAL reductions) and dichotomous initial clinical parameters. The results were expressed as estimate ± standard error (SE) and odds ratio (OR) with 95% confidence interval (CI), respectively. The cut-off values for the initial clinical parameters were 7 mm of PPD, 2 mm of REC, 7 mm of CAL, 2 mm of KM, Grade II mobility, and Degree II FI. The data in GLM were adjusted by age, gender, operator experience (predoctoral and postgraduate levels), GBI, location of tooth, initial FI, and initial adjacent tooth absence or extraction status. All analyses were performed using the Statistical Package for the Social Sciences (SPSS) version 15.0 (IBM Corp., Armonk, NY, USA).

## 3. Results

### 3.1. General Clinical Characteristics

After excluding patients not fulfilling the inclusion criteria or with incomplete records, the charting forms of 204 patients (100 males aged 53.96 ± 12.70 years and 104 females aged 54.02 ± 13.97 years) were included, and a total of 1058 molar teeth with 2773 interproximal sites of ≥5 mm CAL were investigated. At baseline, 778 molar teeth revealed at least one missing adjacent tooth (187 teeth revealed missing adjacent teeth at both sides), and 840 sites revealed no adjacent teeth. During NSPT, 32 examined teeth and 64 examined sites experienced one adjacent tooth extraction.

### 3.2. Initial and Change Data of Low and High Responders (LR and HR)

The initial data of examined teeth and sites, dichotomized by the cut-off values of PPD reduction and CAL gain after NSPT, are shown in Table 1. At the tooth level, HR (*n* = 529) revealed significantly greater initial PPD (*p* < 0.001 by PPD reduction, and *p* < 0.01 by CAL reduction) and lesser initial REC (*p* < 0.001) relative to LR (*n* = 529). At the site level, HR (*n* = 1386) revealed significantly greater initial PPD, CAL, and lesser REC relative to LR (*n* = 1387) (*p* < 0.001 for all). In addition to PPD and CAL reductions, by the dichotomization of PPD reduction, HR showed significantly greater REC gain (*p* < 0.001), KM gain (*p* < 0.01), and mobility reduction (*p* < 0.05) relative to those of LR. Dichotomized by CAL reduction, HR showed significantly lesser REC gain (*p* < 0.001) and greater mobility reduction (*p* < 0.05).

### 3.3. The Status of Adjacent Teeth on the Changes of Site-Level Clinical Parameters

The changes in clinical parameters of the sites with ≥5 mm CAL are shown in Table 2. PPD and CAL reductions were significantly greater (*p* < 0.01 for both) when the adjacent tooth was present at baseline. Among the sites with adjacent teeth at baseline, PPD and CAL reductions were significantly greater (*p* < 0.001 for both) in those that experienced subsequent adjacent tooth extraction.

### 3.4. The Correlation between Adjacent Tooth Status and Better Clinical Outcome of NSPT

The correlations between initial clinical parameters and better clinical outcomes of all examined sites (greater PPD or CAL reduction) were evaluated by GLM and logistic regression and are shown in Table 3. Interproximal sites with initial ≥7 mm PPD, <2 mm REC, ≥7 mm initial CAL, or <Degree II initial FI had a significantly greater tendency to achieve greater PPD and CAL reductions after NSPT. In proximal sites without adjacent teeth, greater PPD reduction (0.23 ± 0.06 mm; OR 1.39, CI 1.07–1.82) and CAL reduction (0.22 ± 0.07 mm; OR 1.22, 0.93–1.61) were observed.

The correlations between initial clinical parameters and better clinical outcomes of the sites with initial presence of adjacent teeth are shown in Table 4. Sites with parameters including ≥7 mm initial PPD, <2 mm REC, ≥7 mm initial CAL, ≥2 mm KM, <Degree II initial FI, and location at the lingual or palatal surface of the tooth showed a significantly greater tendency to achieve greater PPD and CAL reductions after NSPT. When an adjacent tooth was extracted during NSPT, greater PPD reduction (0.60 ± 0.17 mm; OR 2.55, CI 1.08–6.05) and CAL reduction (0.83 ± 0.20 mm; OR 1.51, CI 0.65–3.50) were observed.

## 4. Discussion

The rationale of NSPT is to remove all etiological factors in order to prevent further loss of supporting tissue and achieve a maintainable environment for proper oral hygiene regimens. PPD reduction and CAL reduction have been frequently assessed as outcome parameters of NSPT [17,18,19]. As described in our previous publication [20], 1.64 ± 0.57 mm PPD reduction and 1.25 ± 0.60 mm CAL reduction were achieved in the periodontitis-affected sites of entire dentitions from the same study population after NSPT, and this range of improvement was in agreement with the literature from Cobb [21], indicating that a 1–3 mm PPD reduction and a 0–2 mm CAL reduction could be expected.

The improvement of the interproximal sites on molars, as reported in this study (Table 1), was inferior to that of entire dentitions [20]. This phenomenon is potentially associated with inferior cleaning effectiveness [22,23]. Due to the complexity of root anatomy on molars, proper oral hygiene was not always achievable, leading to more pronounced periodontal inflammation compared with non-molars [18].

Silness et al. [24] reported that the loss of distal teeth was associated with improved periodontal condition of premolars. A major influence on adjacent teeth is the potential for food impaction, which has been recognized as a major extrinsic factor causing periodontal and peri-implant inflammation and interproximal bone loss [25,26]. The determinable factors of food impaction are the alignment and proximity of teeth, as well as the presence of FI [27]. When an adjacent tooth was absent, the anatomical undercuts trapping food debris between the teeth were missing. Access to the interproximal surfaces could be easily established to result in better root instrumentation, and plaque retention and inflammation were possibly reduced. The data from this study revealed that the absence or extraction of a tooth adjacent to periodontitis-affected molars during NSPT contributed to improved treatment outcomes (greater PPD and CAL reductions) (Table 3 and Table 4) and supported the argument that the absence of adjacent teeth could provide additional benefits to NSPT on molars.

Although studies have not suggested tooth type as a major determinant of NSPT efficacy, their results have still revealed that cleaning efficiency and tissue reattachment were apparently lower on molars, specifically for the sites affected by FI [22,23,28]. The present study also reported that Degree II initial FI negatively influenced the outcome of NSPT, regardless of the adjacent tooth status (OR 0.51–0.63; Table 3 and Table 4). The compromised healing in the sites affected by FI was related to poor accessibility, specifically at the furcation fornix, as well as concavities and irregularity presented on the inner surface of roots. FI on the interproximal surfaces may also limit adequate plaque control and accelerate microbial recolonization subgingivally [28,29,30].

The results from this study revealed that better clinical outcomes were generally achievable at the sites with initial greater severity of periodontal destruction (Table 3 and Table 4). This finding that NSPT is effective in treating initial severe periodontitis, is in agreement with the previous literature [17]. This phenomenon is associated with the difficulty of plaque control. As reported by Waerhaug [31], successful plaque control was achieved in only 11% of teeth with >5 mm PPD. Thus, sites with deeper PPD might greatly benefit from professional ScRP compared with those with shallow PPD. A previous study by Badersten et al. [17] also found that PPD and CAL reductions were greater for surfaces with 6–7.5 mm PPD than those with 4–5.5 mm PPD.

The reduction of PPD after NSPT is partially attributed to the shrinkage of gingival apparatus following the reduction of gingival inflammation [32]. Our previous study confirmed that sites with lesser REC are associated with larger shrinkage of soft tissue after NSPT [33]. Lesser REC is also correlated with larger KM (>3 mm) and attached gingiva (>1 mm), which serve as barriers to inflammation and minimize mechanical trauma. Taken together, sites with lesser initial REC were prone to result in greater PPD or CAL reduction after NSPT. The results of the present study also revealed that lingual or palatal sites had a greater potential to achieve PPD or CAL reduction than buccal sites (Table 3 and Table 4). This may be due to poor initial hygiene status in these areas because of the difficulty of self-cleaning, with professional cleaning thus providing a greater chance to access these areas.

Overall, better outcomes of NSPT have been observed at sites with a greater severity of periodontitis, specifically for those with ≥7 mm PPD or ≥7 mm CAL. The absence of adjacent teeth, either at baseline or following extraction during treatment, contributed to better outcomes of NSPT. This may serve as a reference for clinicians contemplating the benefit of extracting a hopeless or strategically non-important tooth during periodontal treatment.

A major limitation of this study was that the reason for tooth extraction and pre-extraction condition were not considered in the analysis. In addition, although more than 2700 sites and 1000 teeth were analyzed in the study, only a few (64 sites in 32 teeth) underwent adjacent tooth extraction. A large-scaled prospective study with a split-mouth design would be still necessary for better understanding the influence of tooth extraction during NSPT.

## 5. Conclusions

Missing or extracting teeth during NSPT resulted in greater PPD and CAL reductions at the adjacent severely periodontitis-affected interproximal sites of molars after NSPT. Thus, removing a hopeless or strategically non-important adjacent tooth could be considered as an integral part of NSPT.

## Figures and Tables

**Table 1:** Tooth and site-level assessments of initial and changes of clinical parameters, including probing pocket depth (PPD), gingival recession (REC), clinical attachment loss (CAL), keratinized mucosa (KM), and tooth mobility, of the initial severe periodontal destruction sites. The median of PPD or CAL change in the severe periodontal destruction sites (initial CAL ≥ 5 mm) of the molar region in the respective individual were used to categorize low (LR) and high-responder (HR). The data were presented as mean ± SD (IQR).

Clinical Parameters		Tooth-Level					Site-Level				
		All	Greater PPD Reduction		Greater CAL Reduction		All	Greater PPD Reduction		Greater CAL Reduction	
			LR	HR	LR	HR		LR	HR	LR	HR
PPD (mm)	Initial	5.29 ± 1.16 (4.6–6)	5.04 ± 1.07 (4–5.4)	5.81 ± 1.17 (5–6.33) ***	5.23 ± 1.15 (4.33–5.67)	5.50 ± 1.19 (5–6) **	5.26 ± 1.77 (4–6)	4.78 ± 1.70 (3–5)	6.03 ± 1.60 (5–7) ***	5.02 ± 1.72 (3–5)	5.87 ± 1.76 (5–7) ***
	Change	1.40 ± 0.99 (0.75–2)	1.02 ± 0.81 (0.33–1)	2.19 ± 0.87 (1.67–2.5)	1.20 ± 0.90 (0.33–1.17)	2.08 ± 1.01 (1.5–2.5)	1.32 ± 1.50 (0–2)	0.60 ± 1.23 (0–1)	2.50 ± 1.13 (2–3) ***	0.89 ± 1.34 (0–1)	2.43 ± 1.33 (2–3) ***
REC (mm)	Initial	1.01 ± 1.13 (0–1.75)	1.28 ± 1.12 (0.6–2)	0.47 ± 0.93 (0–1) ***	1.08 ± 1.13 (0.33–2)	0.79 ± 1.10 (0–1.33) ***	1.25 ± 1.45 (0–2)	1.51 ± 1.48 (0–2)	0.82 ± 1.28 (0–2) ***	1.30 ± 1.43 (0–2)	1.12 ± 1.48 (0–2) ***
	Change	0.38 ± 0.78 (0–0.8)	0.30 ± 0.76 (0–0.6)	0.54 ± 0.79 (0–1)	0.52 ± 0.70 (0–1)	−0.08 ±0.85 (0–0.6)	0.40 ± 1.04 (0–1)	0.28 ± 1.04 (0–1)	0.59 ± 1.00 (0–1) ***	0.58 ± 0.96 (0–1)	−0.07 ± 1.08 (0–1) ***
CAL (mm)	Initial	6.31 ± 1.20 (5.5–7)	6.32 ± 1.18 (5.5–6.8)	6.28 ± 1.23 (5.33–7)	6.31 ± 1.15 (5.5–7)	6.29 ±1.33 (5.33–7)	6.51 ± 1.79 (5–7)	6.30 ± 1.67 (5–7)	6.86 ± 1.93 (5–8) ***	6.32 ± 1.64 (5–7)	6.99 ± 2.05 (5–8) ***
	Change	1.02 ± 1.10 (0.33–1.67)	0.71 ± 1.02 (0–1)	1.65 ± 1.00 (1–2)	0.68 ± 0.90 (0–0.67)	2.16 ± 0.93 (1.33–2)	0.92 ± 1.66 (0–2)	0.32 ± 1.52 (−1–1)	1.91 ± 1.39 (1–2) ***	0.31 ± 1.36 (−1–0)	2.50 ± 1.28 (1–3) ***
KM (mm)	Initial	3.38 ± 1.39 (3–4)	3.34 ± 1.35 (2–4)	3.47 ± 1.46 (3–4)	3.39 ± 1.41 (2–4)	3.37 ± 1.30 (3–4)	3.40 ± 1.39 (3–4)	3.35 ± 1.35 (2–4)	3.47 ± 1.44 (3–4) *	3.40 ± 1.38 (2–4)	3.39 ± 1.40 (3–4)
	Change	0.22 ± 1.13 (−1–1)	0.21 ± 0.94 (0–1)	0.25 ± 1.44 (0–1)	0.22 ± 0.96 (0–1)	0.23 ±1.58 (0–1)	0.22 ± 1.01 (0–1)	0.20 ± 1.02 (0–1)	0.27 ± 1.21 (0–1) **	0.22 ± 1.03 (0–1)	0.23 ± 1.27 (0–1)
Mobility (Grade)	Initial	0.62 ± 1.20 (0–1)	0.71 ± 1.38 (0–1)	0.44 ± 0.67 (0–1)	0.68 ± 1.28 (0–1)	0.44 ± 0.87 (0–1)	0.61 ± 0.74 (0–1)	0.61 ± 0.73 (0–1)	0.60 ± 0.74 (0–1)	0.62 ± 0.73 (0–1)	0.57 ± 0.74 (0–1) *
	Change	−0.05 ± 1.34 (0–1)	-0.06 ± 1.50 (0–1)	−0.03 ± 0.92 (0–1)	−0.06 ± 1.44 (0–1)	−0.03 ± 0.88 (0–1)	−0.09 ± 0.51 (0–0)	−0.07 ± 0.52 (0–0)	−0.12 ± 0.50 (0–0) *	−0.07 ± 0.51 (0–0)	−0.13 ± 0.51 (0–0) **

Significant difference between the low- and high-responder: * *p* < 0.05; ** *p* < 0.01; *** *p* < 0.001.

**Table 2:** The effect of adjacent tooth absence or extraction on the clinical parameter changes, including probing pocket depth (PPD), gingival recession (REC), clinical attachment loss (CAL), keratinized mucosa (KM), and tooth mobility, of initial severe periodontal destruction sites in the molar interproximal region. The data are presented as mean ± SD (IQR).

Clinical Parameters Change	Adjacent Tooth Absence before NSPT		Adjacent Tooth Extraction during NSPT	
	Yes	No	Yes	No
PPD (mm)	1.33 ± 1.46 (0–2)	1.49 ± 1.46 (0–2) **	2.25 ± 1.57 (1–3)	1.47 ± 1.44 (0–2) ***
REC (mm)	0.45 ± 1.03 (0–1)	0.43 ± 1.05 (0–1)	0.25 ± 1.67 (0–1)	0.44 ± 1.03 (0–1)
CAL (mm)	0.89 ± 1.57 (0–2)	1.06 ± 1.68 (0–2) **	2.00 ± 2.30 (1–3)	1.03 ± 1.64 (0–2) ***
KM (mm)	0.19 ± 1.34 (0–1)	0.23 ± 1.06 (0–1)	0.34 ± 0.96 (0–1)	0.22 ± 1.06 (0–1)
Mobility (Grade)	−0.09 ± 0.54 (0–0)	−0.09 ± 0.49 (0–0)	−0.16 ± 0.52 (0–0)	−0.08 ± 0.49 (0–0)

Significant differences among tooth missing or extraction status: ** *p* < 0.01; *** *p* < 0.001. NSPT: non-surgical periodontal treatment.

**Table 3:** Multivariate analysis (MA) and odds ratio (OR) of the initial clinical factors affecting the treatment outcomes of initial severe periodontal destruction sites in the molar interproximal region. The parameters to assess the treatment outcomes were the mean probing pocket depth (PPD) and clinical attachment loss (CAL) changes of these sites in individuals. The multivariate analysis was performed using a generalized linear model with the adjustments of age, gender, operator experience, gingival bleeding index, location of tooth, position of site, initial furcation involvement, and initial adjacent tooth missing status. The data are presented as estimate ± standard errors. OR was determined by logistic regression, and the data are presented as OR (95% confidence interval). Gingival recession (REC); keratinized mucosa (KM); furcation involvement (FI); not applicable (NA).

Factors Examined	All Interproximal Sites			
	Greater PPD Reduction		Greater CAL Reduction	
	MA	OR	MA	OR
7 mm initial PPD	0.70 ± 0.08 ***	1.67 (1.15–2.42) **	0.34 ± 0.10 **	1.01 (0.69–1.49)
2 mm initial REC	−0.74 ± 0.06 ***	0.35 (0.26–0.48) ***	−0.20 ± 0.08 **	0.66 (0.48–0.90) **
7 mm initial CAL	0.57 ± 0.08 ***	2.16 (1.51–3.09) ***	0.64 ± 0.09 ***	2.00 (1.39–2.88) ***
2 mm initial KM	0.27 ± 0.11.*	0.84 (0.50–1.41)	0.23 ± 0.13	0.64 (0.38–1.07)
Gr II initial mobility	−0.12 ± 0.09	1.00 (0.68–1.46)	−0.38 ± 0.10 ***	0.81 (0.54–1.21)
Degree II initial FI	NA	0.63 (0.48–0.83) **	NA	0.61 (0.46–0.81) **
Maxillary vs. mandibular	NA	1.15 (0.91–1.45)	NA	1.62 (1.27–2.07) ***
Buccal vs. lingual (palatal)	NA	0.79 (0.63–0.99) *	NA	0.83 (0.66–1.05)
First molar vs. Second molar	NA	0.96 (0.75–1.24)	NA	1.23 (0.95–1.60)
Postgraduate vs. predoctoral level	NA	0.71 (0.55–0.93) *	NA	0.80 (0.61–1.06)
Adjacent tooth missing before NSPT	0.23 ± 0.06 ***	1.39 (1.07–1.82) *	0.22 ± 0.07 **	1.22 (0.93–1.61)

Significant differences among tooth missing or extraction status: * *p* < 0.05; ** *p* < 0.01; *** *p* < 0.001.

**Table 4:** Multivariate analysis (MA) and odds ratio (OR) of the clinical factors affecting the treatment outcomes of initial severe periodontal destruction sites in the molar interproximal sites without absent adjacent tooth before NSPT. The parameters to assess the treatment outcome were the mean probing pocket depth (PPD) and clinical attachment loss (CAL) changes of these sites in individuals. The multivariate analysis was performed using a generalized linear model with the adjustments of age, gender, operator experience, gingival bleeding index, location of tooth, position of site, initial furcation involvement, and the adjacent tooth extraction status. The data are presented as estimate ± standard errors. OR was determined by logistic regression, and the data are presented as OR (95% confidence interval). Gingival recession (REC); keratinized mucosa (KM); furcation involvement (FI); not applicable (NA).

Factors Examined	Interproximal Sites without Missing Adjacent Tooth before NSPT			
	Greater PPD Reduction		Greater CAL Reduction	
	MA	OR	MA	OR
7 mm initial PPD	0.80 ± 0.10 ***	1.80 (1.11–2.90) *	0.42 ± 0.12 **	0.91 (0.56–1.49)
2 mm initial REC	−0.79 ± 0.07 ***	0.29 (0.20–0.43) ***	−0.18 ± 0.09 *	0.64 (0.44–0.93) *
7 mm initial CAL	0.52 ± 0.09 ***	2.07 (1.33–3.23) **	0.65 ± 0.11 ***	2.25 (1.45–3.50) ***
2 mm initial KM	−0.41 ± 0.15 **	0.79 (0.39–1.61)	−0.37 ± 0.18 *	0.58 (0.29–1.18)
Gr II initial mobility	0.12 ± 0.11	1.66 (1.01–2.74) *	−0.32 ± 0.13 *	1.09 (0.66–1.82)
Degree II initial FI	NA	0.51 (0.37–0.72) ***	NA	0.54 (0.38–0.77) **
Maxillary vs. mandibular	NA	1.12 (0.84–1.49)	NA	1.81 (1.35–2.43) ***
Buccal vs. lingual (palatal)	NA	0.75 (0.57–0.98) *	NA	0.74 (0.56–0.99) *
First molar vs. second molar	NA	0.87 (0.65–1.16)	NA	1.11 (0.82–1.50)
Postgraduate vs. predoctoral level	NA	0.61 (0.44–0.84) **	NA	0.78 (0.56–1.08)
Adjacent tooth extraction during NSPT	0.60 ± 0.17 ***	2.55 (1.08–6.05) *	0.83 ± 0.20 ***	1.51 (0.65–3.50)

Significant differences among tooth missing or extraction status: * *p* < 0.05; ** *p* < 0.01; *** *p* < 0.001. NSPT: non-surgical periodontal treatment.
